# Distinct Cdk1 Requirements during Single-Strand Annealing, Noncrossover, and Crossover Recombination

**DOI:** 10.1371/journal.pgen.1002263

**Published:** 2011-08-25

**Authors:** Camilla Trovesi, Marco Falcettoni, Giovanna Lucchini, Michela Clerici, Maria Pia Longhese

**Affiliations:** Dipartimento di Biotecnologie e Bioscienze, Università di Milano-Bicocca, Milano, Italy; Fred Hutchinson Cancer Research Center, United States of America

## Abstract

Repair of DNA double-strand breaks (DSBs) by homologous recombination (HR) in haploid cells is generally restricted to S/G2 cell cycle phases, when DNA has been replicated and a sister chromatid is available as a repair template. This cell cycle specificity depends on cyclin-dependent protein kinases (Cdk1 in *Saccharomyces cerevisiae*), which initiate HR by promoting 5′–3′ nucleolytic degradation of the DSB ends. Whether Cdk1 regulates other HR steps is unknown. Here we show that *yku70Δ* cells, which accumulate single-stranded DNA (ssDNA) at the DSB ends independently of Cdk1 activity, are able to repair a DSB by single-strand annealing (SSA) in the G1 cell cycle phase, when Cdk1 activity is low. This ability to perform SSA depends on DSB resection, because both resection and SSA are enhanced by the lack of Rad9 in *yku70Δ* G1 cells. Furthermore, we found that interchromosomal noncrossover recombinants are generated in *yku70Δ* and *yku70Δ rad9Δ* G1 cells, indicating that DSB resection bypasses Cdk1 requirement also for carrying out these recombination events. By contrast, *yku70Δ* and *yku70Δ rad9Δ* cells are specifically defective in interchromosomal crossover recombination when Cdk1 activity is low. Thus, Cdk1 promotes DSB repair by single-strand annealing and noncrossover recombination by acting mostly at the resection level, whereas additional events require Cdk1-dependent regulation in order to generate crossover outcomes.

## Author Summary

Homologous recombination (HR) provides an important mechanism to eliminate deleterious lesions, such as DNA double-strand breaks (DSBs). DSB repair by HR uses homologous DNA sequences as a template to form recombinants that are either crossover or noncrossover with regard to flanking parental sequences. Furthermore, a DSB flanked by direct DNA repeats can be repaired by another HR pathway called single-strand annealing (SSA). HR is generally confined to the S and G2 phases of the cell cycle, when DNA has been replicated and a sister chromatid is available as repair template. This cell cycle specificity depends on the activity of cyclin-dependent kinases (Cdks), which regulate initiation of HR by promoting nucleolytic degradation (resection) of the DSB ends. Whether Cdks regulate other HR steps is unknown. Here, we show that *Saccharomyces cerevisiae* Cdk1 has a dual function in HR: it promotes SSA and noncrossover recombination by regulating primarily the resection step, whereas it plays additional functions in allowing recombination accompanied by crossovers. As crossovers during mitotic cell growth have the potential for deleterious genome rearrangements when the sister chromatid is not used as repair template, this additional function of Cdk1 in promoting crossovers can provide another safety mechanism to ensure genome stability.

## Introduction

DNA double-strand breaks (DSBs) occur spontaneously during DNA replication and after exposure to certain genotoxic chemicals or ionizing radiation. Efficient repair of DSBs can be accomplished by nonhomologous end joining (NHEJ), which directly rejoins broken DNA ends, or by homologous recombination (HR), which utilizes a homologous DNA template to restore the genetic information lost at the break site (reviewed in [1]–[3]). Failure to repair DSBs can lead to genome instability and cell death.

HR is initiated by 5′-3′ nucleolytic degradation of the DSB ends to yield 3′-ended single-stranded DNA (ssDNA) tails. Replication protein A (RPA) binds to the ssDNA tails to remove their secondary DNA structures, but is then replaced by Rad51 aided by Rad52. Once formed, the Rad51 nucleofilaments search for homologous sequences and then promote invasion of the ssDNA into homologous donor double-stranded DNA to form a joint molecule with a displaced strand (D-loop) (reviewed in [1]–[3]). Following strand invasion, the 3′ end of the invading strand primes DNA synthesis using the donor sequence as a template, thus restoring those residues that were lost by resection [4].

According to the canonical double-strand break repair (DSBR) model [5], the displaced strand of the D-loop can anneal with the complementary sequence on the other side of the break (second end capture) to form a double Holliday junction (dHJ) intermediate. Random cleavage of the two HJs is expected to yield an equal number of noncrossover and crossover products. This DSBR model predicts that both crossover and noncrossover products derive from dHJ resolution. However, the finding that most DSB repair in somatic cells is not associated with crossovers [6] led to alternative models for noncrossover generation. In one of them, the action of helicases mediates the convergent branch migration of the two HJs, thus producing a hemicatenane structure that is decatenated to form exclusively noncrossover products [7]–[9]. A second mechanism, termed synthesis-dependent strand annealing (SDSA), leads to displacement of the invading strand that has been extended by DNA synthesis and that anneals with the complementary sequences exposed by 5′-3′ resection [10]–[12]. Because no HJ is formed, only noncrossover products are made. Interestingly, during meiotic recombination, where dHJ resolution into crossovers is essential to drive segregation of homologs to opposite poles, most crossovers are thought to arise via dHJ resolution, whereas noncrossovers form mostly by the SDSA pathway [13], [14].

When a DSB is flanked by direct repeats, its repair primarily occurs by single-strand annealing (SSA). Here, the resected DSB ends anneal with each other instead of invading a homologous DNA sequence (reviewed in [1]–[3]). Subsequent nucleolytic removal of the protruding single-stranded tails results in deletion of the intervening DNA sequence and one of the repeats. In principle, such a break can also be repaired by break-induced replication (BIR), where the repeat closer to the cut site can strand-invade the repeat that is further away and set up a recombination-dependent replication fork to copy all the distal sequences. However, SSA usually out-competes BIR, which is a kinetically slow process [15].

All the above HR pathways require 5′-3′ nucleolytic degradation of DNA ends and the strand-annealing activity of Rad52. In addition, DSBR, SDSA and BIR require the Rad51 protein, which is dispensable for SSA that does not involve strand invasion [16].

In *Saccharomyces cerevisiae* haploid cells, mitotic HR is generally restricted to the S and G2 phases of the cell cycle, when DNA has been replicated and a sister chromatid is available as an appropriate donor [17], [18]. This cell-cycle specificity depends on cyclin-dependent kinases (Cdks; Cdk1 in *S. cerevisiae*), which promote resection of the 5′ DSB ends to yield 3′-ended ssDNA tails that are necessary to initiate HR [17], [18]. End resection occurs through a biphasic mechanism: first the MRX complex and Sae2 clip 50–100 nucleotides from the 5′ DNA ends; then Exo1 or Sgs1-Top3-Rmi1 and Dna2 process the early intermediate to form extensive regions of ssDNA (reviewed in [19], [20]). The Sae2 protein has been shown to be a Cdk1 target in promoting ssDNA generation at DNA ends during both mitosis and meiosis [21], [22]. However, as Sae2 only resects a relatively small amount of DNA and other nucleases and helicases are required for efficient DSB resection, Cdk1 likely has additional targets in promoting this event.

Indeed, DSB end resection is also negatively regulated by the Yku heterodimer [23], [24] and by the checkpoint protein Rad9 [25], [26]. Interestingly, the ends of an endonuclease-induced DSB are resected in the G1 phase of the cell cycle (low Cdk1 activity) when Yku is lacking [24]. Moreover, *RAD9* deletion allows DSB resection in G2 cells that display low Cdk1 activity due the overexpression of the Cdk1 inhibitor Sic1 [26]. These findings indicate that Cdk1 requirement for DSB resection is bypassed when the inhibitory function of either Yku or Rad9 is relieved.

Whether Cdk1 promotes other HR events is unknown. Some evidence suggests that HR steps other than DSB resection might be regulated by Cdk1 activity. For example, formation of Rad52 foci after ionizing radiation (IR) is less efficient in G1 than in G2, suggesting that Cdk1 might control Rad52 recruitment to DSBs [27]. Furthermore, Cdk1 targets the Srs2 helicase to dismantle D-loop structures, possibly by counteracting unscheduled Srs2 sumoylation [28]. Proteins implicated in late HR events have also been identified as potential Cdk substrates in other eukaryotes. In particular, human BRCA2 is phosphorylated by Cdks, and this phosphorylation has been proposed to negatively regulate Rad51 recombination activity [29]. Moreover, Cdk1-dependent phosphorylation of the fission yeast checkpoint protein Crb2 stimulates resolution of HR intermediates by the topoisomerase Top3 and the ReqQ helicase Rqh1 [30].

Here, we investigate the role of Cdk1 in homology-dependent repair of a DSB. We show that generation of 3′-ended ssDNA at the DSB ends bypasses Cdk1 requirement for the repair of a DSB by either SSA or noncrossover recombination, indicating that Cdk1 is dispensable for these repair events if DSB resection occurs. By contrast, resection is not sufficient to bypass Cdk1 requirement for generating crossover products. Thus, Cdk1 promotes SSA- and noncrossover-mediated recombination by regulating essentially the resection step, while Cdk1 controls further HR steps in order to allow crossover outcomes.

## Results

### The lack of Yku70 allows DSB repair by SSA in G1

HR is inhibited in G1 when Cdk1 activity is low, whereas it occurs during S and G2/M cell cycle phases when Cdk1 activity is high [17], [18]. Although it is well known that Cdk1 promotes resection of DSB ends [17], [18], [21], it is still unclear if other HR steps are regulated by Cdk1. To investigate whether DSB resection is the only step controlled by Cdk1 in HR-mediated DSB repair, we asked if generation of ssDNA at the DSB ends is sufficient to allow HR when Cdk1 activity is low. As DSB resection in G1 is inhibited by the Yku heterodimer and *YKU70* deletion allows ssDNA generation at DSB ends in G1 cells [24], we asked if *yku70Δ* cells are capable to carry out HR in G1.

**Figure 1:**
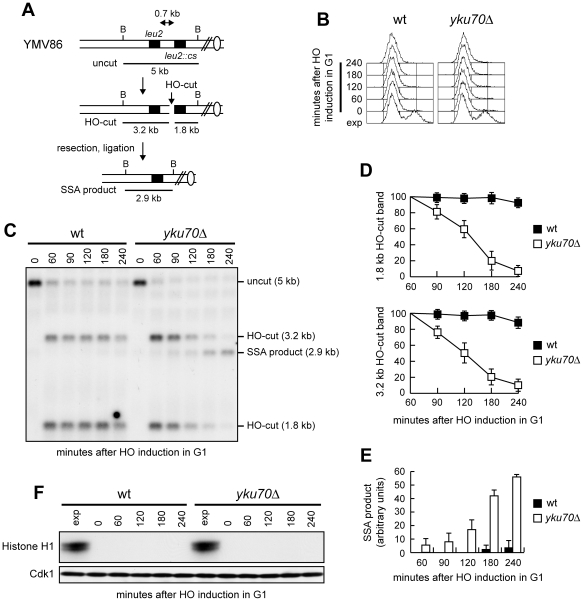
SSA-mediated DSB repair in *yku70Δ* G1 cells. (A) Map of the YMV86 chromosome III region where the HO-cut site is flanked by homologous *leu2* sequences that are 0.7 kb apart. HO-induced DSB formation results in generation of 3.2 kb and 1.8 kb DNA fragments (HO-cut) that can be detected by Southern blot analysis of BglII-digested genomic DNA with a *LEU2* probe. DSB repair by SSA generates a product of 2.9 kb (SSA product). B, BglII. (B–E) Exponentially growing YEP+raf (exp) cell cultures of wild type YMV86 and its *yku70Δ* derivative strain were arrested in G1 with α-factor (time zero) and transferred to YEP+raf+gal in the presence of α-factor. (B) FACS analysis of DNA content. (C) Southern blot analysis of BglII-digested genomic DNA. (D, E) Densitometric analysis of the HO-cut (D) and the SSA (E) band signals. Plotted values are the mean value ±SD from four independent experiments as in (C), enclosing that described in (F). The intensity of each band was normalized with respect to a loading control. (F) YMV86 derivative strains with the indicated genotypes and expressing fully functional Cdc28-HA were treated as in (B–E). Cell samples were collected at the indicated times to assay Cdk1 kinase activity in anti-HA immunoprecipitates by using histone H1 as substrate (top row) and to determine Cdk1 levels by western blot analysis with anti-HA antibody (bottom row).

Homology-dependent repair of a DSB made between tandem DNA repeats occurs primarily by SSA [15], which requires DSB resection and re-annealing of RPA-covered ssDNA by the Rad52 protein [1], [31]. This process does not involve strand invasion and is therefore independent of Rad51 [16]. We deleted *YKU70* in a strain where tandem repeats of the *LEU2* gene are 0.7 kb apart and one of them (*leu2::cs*) is adjacent to a recognition site for the HO endonuclease (Figure 1A) [32]. The strain also harbors a *GAL-HO* construct that provides regulated *HO* expression. Since homology is restricted to only one DSB end (Figure 1A), the HO-induced break cannot be repaired by gene conversion, making SSA the predominant repair mode. HO was expressed by galactose addition to α-factor-arrested cells that were kept arrested in G1 with α-factor for the subsequent 4 hours. Galactose was maintained in the medium in order to permanently express HO, which can recurrently cleave the HO sites eventually reconstituted by NHEJ-mediated DSB repair. Kinetics of DSB repair was evaluated by Southern blot analysis with a *LEU2* probe that also allowed following 5′-end resection on each side of the break by monitoring the disappearance of the HO-cut DNA bands. The quality and persistence of the cell cycle arrest was assessed by FACS analysis (Figure 1B) and by measuring Cdk1 kinase activity (Figure 1F). Consistent with the requirement of Cdk1 activity for DSB resection and repair, both the 1.8 kb and 3.2 kb HO-cut band signals remained high throughout the experiment in wild type G1 cells (Figure 1C and 1D), where the 2.9 kb SSA repair product was only barely detectable (Figure 1C and 1E). By contrast, the SSA repair product accumulated in *yku70Δ* G1 cells (Figure 1C and 1E), where both the 1.8 kb and 3.2 kb HO-cut band signals decreased (Figure 1C and 1D). The ability of *yku70Δ* cells to carry out SSA does not require Cdk1. In fact, Cdk1 activity, which was present in exponentially growing wild type and *yku70Δ* cells, dropped to undetectable levels after G1 arrest (time 0) and remained undetectable in both cultures throughout the experiment (Figure 1F). Thus, the lack of Yku allows DSB repair by SSA in G1, suggesting that ssDNA generation is sufficient to bypass Cdk1 requirement for SSA.

**Figure 2:**
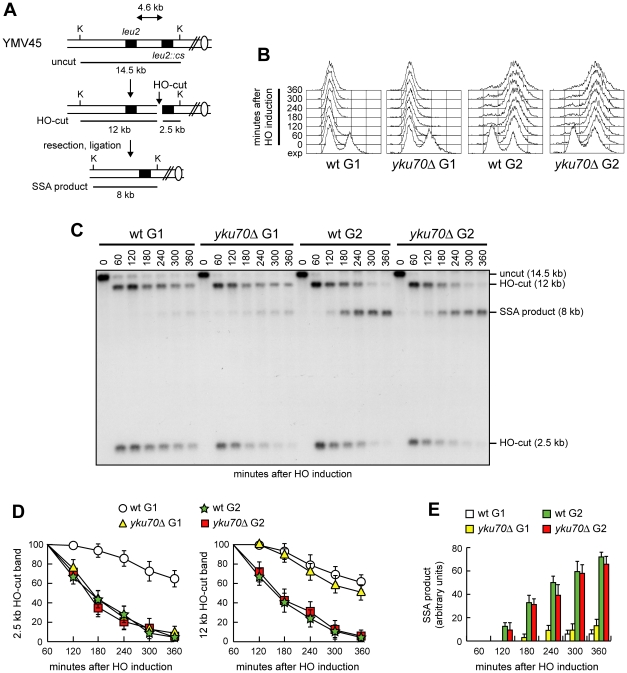
SSA-mediated DSB repair in *yku70Δ* G1 and G2 cells. (A) Map of the YMV45 chromosome III region where the HO-cut site is flanked by homologous *leu2* sequences that are 4.6 kb apart. HO-induced DSB formation results in generation of 12 kb and 2.5 kb DNA fragments (HO-cut) that can be detected by Southern blot analysis of KpnI-digested genomic DNA with a *LEU2* probe. DSB repair by SSA generates a product of 8 kb (SSA product). K, KpnI. (B–E) Exponentially growing YEP+raf (exp) cell cultures of wild type YMV45 and its *yku70Δ* derivative strain were arrested at time zero in G1 with α-factor or in G2 with nocodazole and transferred to YEP+raf+gal in the presence of α-factor or nocodazole, respectively. (B) FACS analysis of DNA content. (C) Southern blot analysis of KpnI-digested genomic DNA. (D, E) Densitometric analysis of the HO-cut (D) and the SSA (E) band signals. Plotted values are the mean value ±SD from three independent experiments as in (C). The intensity of each band was normalized with respect to a loading control.

SSA-based DNA repair requires degradation of the 5′ DSB ends to reach the complementary DNA sequences that can then anneal. If SSA in *yku70Δ* G1 cells depends on generation of 3′-ended ssDNA at DSB ends, then failure of resection to reach the homologous distal *leu2* sequence should prevent SSA. Interestingly, Cdk1-independent resection takes place in *yku70Δ* cells, but it is confined to DNA regions closed to the DSB site [24], suggesting that other proteins limit extensive DSB resection in the absence of Yku. We therefore asked whether increasing the distance between the complementary *leu2* sequences prevented DSB repair by SSA in *yku70Δ* G1 cells. To this end, we monitored SSA-mediated repair of an HO-induced DSB in a strain where the donor *leu2* sequence was positioned 4.6 kb away from the HO recognition site at *leu2::cs* (Figure 2A) [32]. HO expression was induced in α-factor-arrested cells that were kept blocked in G1 with α-factor in the presence of galactose (Figure 2B). Consistent with previous findings [24], resection in *yku70Δ* G1 cells was restricted to DNA regions closed to the break site. In fact, the 2.5 kb HO-cut signal decreased more efficiently in *yku70Δ* than in wild type G1 cells, whereas similar amounts of the 12 kb HO-cut signal were detectable in both wild type and *yku70Δ* G1 cells (Figure 2C and 2D). Thus, 5′-3′ nucleolytic degradation in *yku70Δ* G1 cells failed to proceed beyond the distal *leu2* hybridization region. The inability of resection to uncover the homologous distal *leu2* sequence prevented DSB repair by SSA in *yku70Δ* G1 cells. In fact, the 8 kb SSA repair product was only barely detectable in both wild type and *yku70Δ* G1 cells throughout the experiment (Figure 2C and 2E). By contrast, when a similar experiment was performed in G2-arrested cells (Figure 2B), where the inhibitory function of Yku on DSB resection is relieved [33], [34], the 8 kb SSA repair product was clearly detectable in wild type and *yku70Δ* cells (Figure 2C and 2E), which both showed also a decrease of the 12 kb HO-cut signals compared to the same strains arrested in G1 (Figure 2C and 2D). Thus, the ability of *yku70Δ* G1 cells to repair a DSB by SSA depends on the extent of resection.

### The lack of Rad9 enhances resection in *yku70Δ* G1 cells

**Figure 3:**
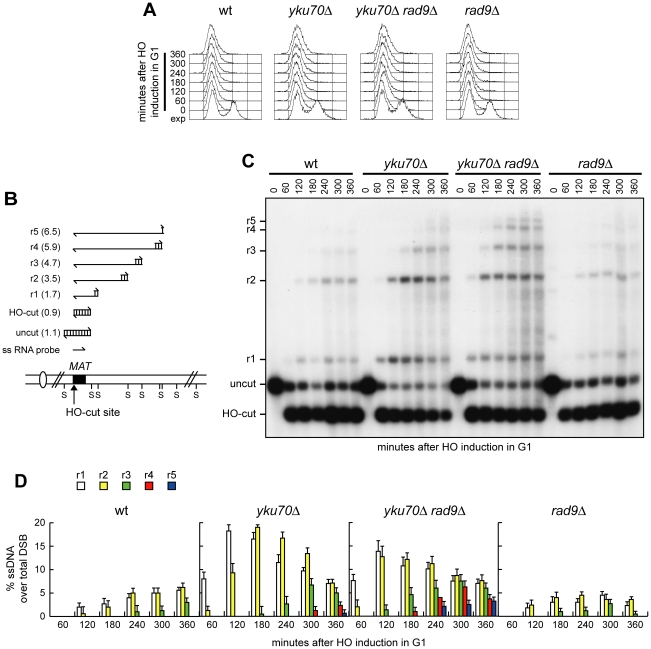
Rad9 inhibits extensive DSB resection in *yku70Δ* G1 cells. Exponentially growing YEP+raf (exp) cell cultures of wild type JKM139 and its derivative mutant strains were arrested in G1 with α-factor (time zero) and transferred to YEP+raf+gal in the presence of α-factor. (A) FACS analysis of DNA content. (B) System used to detect DSB resection. Gel blots of SspI-digested genomic DNA separated on alkaline agarose gel were hybridized with a single-stranded *MAT* probe specific for the unresected strand. 5′-3′ resection progressively eliminates SspI sites (S), producing larger SspI fragments (r1 through r5) detected by the probe. (C) Analysis of ssDNA formation as described in (B). (D) Densitometric analysis of the resection products. Plotted values are the mean value ±SD from three independent experiments as in (C). See Materials and Methods for details.

If ssDNA generation were the limiting step in SSA-mediated DSB repair in G1, then increasing the efficiency/extent of resection should enhance the ability of *yku70Δ* cells to carry out SSA in G1. The lack of the checkpoint protein Rad9 has been shown to allow DSB resection in G2 cells that displayed low Cdk1 activity due to high levels of the Cdk1 inhibitor Sic1 [26]. Thus, we asked whether the lack of Rad9 enhanced the efficiency of DSB resection in *yku70Δ* G1 cells. To compare resection efficiency independently of DSB repair, we monitored the appearance of the resection products at an HO-induced DSB generated at the *MAT* locus (Figure 3B) of G1-arrested (Figure 3A) cells, which were not able to repair this DSB because they lacked the homologous donor sequences *HML* and *HMR* [23]. As expected, wild type cells showed very low levels of the 3′-ended resection products (r1 to r5), which instead clearly accumulated in both *yku70Δ* and *yku70Δ rad9Δ* cells (Figure 3C and 3D). Moreover, the longest r4 and r5 resection products were detectable in *yku70Δ rad9Δ* cells 120 minutes earlier than in *yku70Δ* cells (Figure 3C and 3D), indicating that the lack of Rad9 enhances the resection efficiency of *yku70Δ* G1 cells. Interestingly, although *RAD9* deletion was shown to allow MRX-dependent ssDNA generation in Sic1 overproducing G2 cells [26], *rad9Δ* G1 cells did not show increased efficiency of DSB resection compared to wild type cells (Figure 3C and 3D). Thus, Rad9 limits extensive resection in *yku70Δ* cells, but its lack is not sufficient, by itself, to escape the inhibitory effect of Yku on DSB resection in G1.

### The lack of Rad9 enhances SSA in *yku70Δ* G1 cells

**Figure 4:**
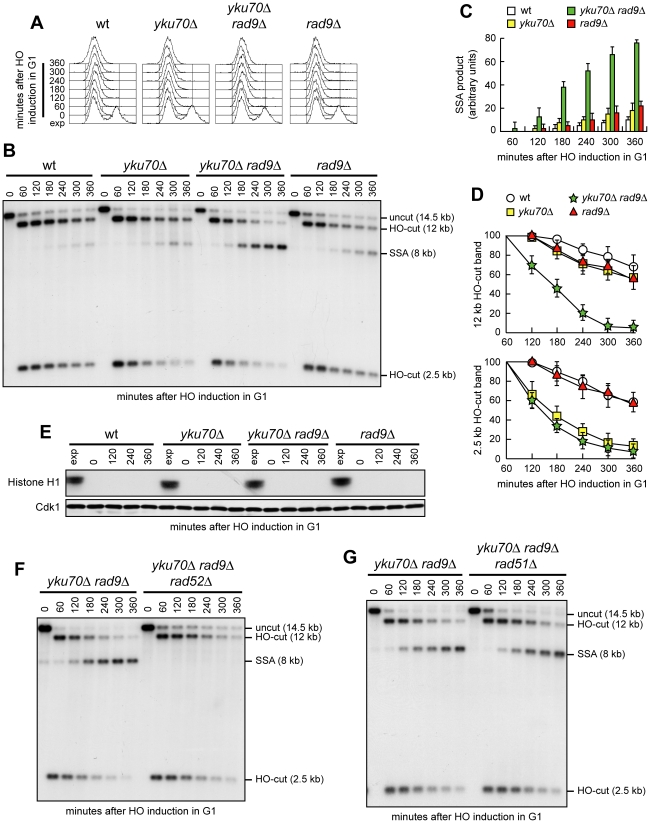
*RAD9* deletion increases SSA efficiency in *yku70Δ* G1 cells. (A–D) Exponentially growing YEP+raf (exp) cell cultures of wild type YMV45 and its derivative mutant strains were arrested in G1 with α-factor (time zero) and transferred to YEP+raf+gal in the presence of α-factor. (A) FACS analysis of DNA content. (B) DSB repair by SSA was analyzed as described in Figure 2. (C, D) Densitometric analysis of the SSA (C) and the HO-cut (D) band signals. Plotted values are the mean value ±SD from four independent experiments as in (B), enclosing that described in (E). (E) YMV45 derivative strains with the indicated genotypes and expressing fully functional Cdc28-HA were treated as in (A–D). Cell samples were taken at the indicated times to assay Cdk1 kinase activity (top row) and to determine Cdk1 levels (bottom row) as in Figure 1F. (F, G) Exponentially growing YEP+raf cell cultures of YMV45 derivative strains were arrested in G1 with α-factor (time zero) and transferred to YEP+raf+gal in the presence of α-factor. DSB repair by SSA was analyzed as described in Figure 2.

Because DSB resection in G1 was more efficient in *yku70Δ rad9Δ* cells than in *yku70Δ* cells, we asked whether the lack of Rad9 allows efficient SSA-mediated DSB repair in *yku70Δ* G1 cells carrying tandem repeats of the *LEU2* gene 4.6 kb apart. Indeed, the amount of SSA repair products in G1 was much higher in *yku70Δ rad9Δ* cells than in wild type, *yku70Δ* or *rad9Δ* cells (Figure 4A–4C). Consistent with DSB resection being more extensive in *yku70Δ rad9Δ* than in *yku70Δ* G1-arrested cells (Figure 3), the decrease of the 12 kb HO-cut band signal was much more apparent in *yku70Δ rad9Δ* than in *yku70Δ* G1 cells, whereas the 2.5 kb HO-cut band signal decreased with similar kinetics in both G1 cell cultures (Figure 4B and 4D). Cdk1 kinase activity, which was present in all exponentially growing cells, was not required for accumulation of the repair products in *yku70Δ rad9Δ* cells, as it was undetectable in all G1-arrested cell cultures throughout the experiment (Figure 4E).

SSA requires the strand-annealing activity of the Rad52 protein, but it occurs independently of Rad51 [16]. Consistent with the SSA repair mode, formation of the repair products in G1-arrested *yku70Δ rad9Δ* cells was abolished by *RAD52* deletion (Figure 4F), whereas it was unaffected by *RAD51* deletion (Figure 4G). As a DSB flanked by direct repeats could be repaired, at least in principle, also by Rad51-dependent BIR [15], the finding that *yku70Δ rad9Δ* and *yku70Δ rad9Δ rad51Δ* G1 cells accumulated the 8 kb repair product with similar kinetics (Figure 4G) indicates that SSA is responsible for this repair event. Thus, we conclude that the lack of Rad9 increases the ability of *yku70Δ* cells to carry out DSB repair by SSA in G1, likely by enhancing the efficiency of DSB resection.

**Figure 5:**
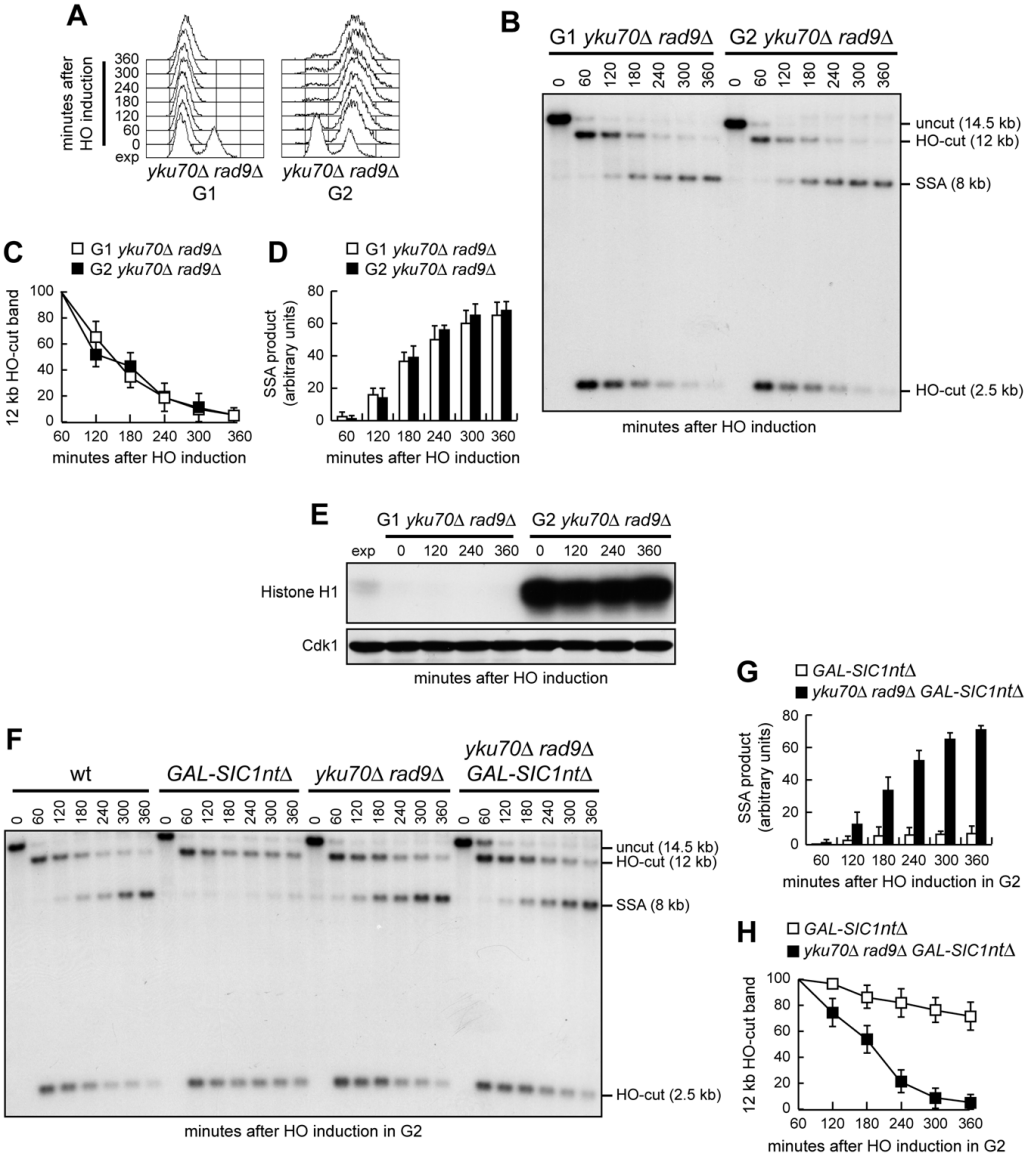
DSB resection is the limiting step in DSB repair by SSA. (A–D) Exponentially growing YEP+raf (exp) YMV45 *yku70Δ rad9Δ* cells were arrested in G1 with α-factor or in G2 with nocodazole and transferred to YEP+raf+gal in the presence of α-factor or nocodazole, respectively. (A) FACS analysis of DNA content. (B) DSB repair by SSA was analyzed as described in Figure 2. (C, D) Densitometric analysis of the 12 kb HO-cut (C) and 8 kb SSA (D) band signals. Plotted values are the mean value ±SD from four independent experiments as in (B), enclosing that described in (E). The intensity of each band was normalized with respect to a loading control. (E) YMV45 *yku70Δ rad9Δ* cells expressing fully functional Cdc28-HA were treated as in (A–D). Cell samples were taken at the indicated times to assay Cdk1 kinase activity (top row) and to determine Cdk1 levels (bottom row) as in Figure 1F. (F–H) Exponentially growing YEP+raf YMV45 derivative cells with the indicated genotypes were arrested at time zero in G2 with nocodazole and transferred to YEP+raf+gal in the presence of nocodazole. Cell cycle arrest was verified by FACS analysis (not shown). (F) DSB repair by SSA was analyzed as described in Figure 2. (G, H) Densitometric analysis of the 8 kb SSA (G) and 12 kb HO-cut (H) band signals. Plotted values are the mean value ±SD from three independent experiments as in (F). The intensity of each band was normalized with respect to a loading control.

If competence for SSA-mediated DSB repair relies solely on 3′-ended ssDNA generation, then this repair process should take place with similar efficiency in G1- and G2-arrested *yku70Δ rad9Δ* cells. As this expectation is based on the assumption that G1- and G2-arrested *yku70Δ rad9Δ* cells resect DSB ends with similar efficiencies, we compared resection (Figure 5B and 5C) and SSA (Figure 5B and 5D) in *yku70Δ rad9Δ* cells arrested either in G1 or in G2 (Figure 5A) during break induction. Disappearance of the 2.5 kb and 12 kb HO-cut bands occurred with similar kinetics in G1- and G2-arrested *yku70Δ rad9Δ* cells (Figure 5B and 5C), which also accumulated similar amounts of the 8 kb SSA repair product (Figure 5B and 5D). As expected, Cdk1 kinase activity was undetectable in *yku70Δ rad9Δ* cells during the α-factor arrest, whereas it was high in nocodazole-arrested G2 cells (Figure 5E). Thus, DSB resection is the limiting step in DSB repair by SSA.

If SSA is generally restricted to G2 only because high Cdk1 activity allows DSB resection, then inactivation of Cdk1 in G2 should prevent SSA in wild type but not in *yku70Δ rad9Δ* cells, where DSB resection occurs independently of Cdk1. Thus, we compared DSB repair by SSA in G2-arrested wild type and *yku70Δ rad9Δ* cells expressing high levels of a stable version of the mitotic Clb-Cdk1 inhibitor Sic1 (Sic1nt*Δ*) [35]. Consistent with the hypothesis that Cdk1 promotes SSA by regulating the resection step, Sic1 overproduction inhibited SSA repair in G2-arrested wild type cells but not in *yku70Δ rad9Δ* cells. In fact, the 8 kb SSA repair product accumulated in *yku70Δ rad9Δ GAL-SIC1ntΔ* cells (Figure 5F and 5G), which showed a decrease of both the 2.5 kb and 12 kb HO-cut band signals (Figure 5F and 5H). By contrast, the same repair product was only barely detectable in G2-arrested *GAL-SIC1ntΔ* cells, where the HO-cut band signals remained high throughout the experiment (Figure 5F–5H).

### The lack of Yku70 allows noncrossover recombination in G1

**Figure 6:**
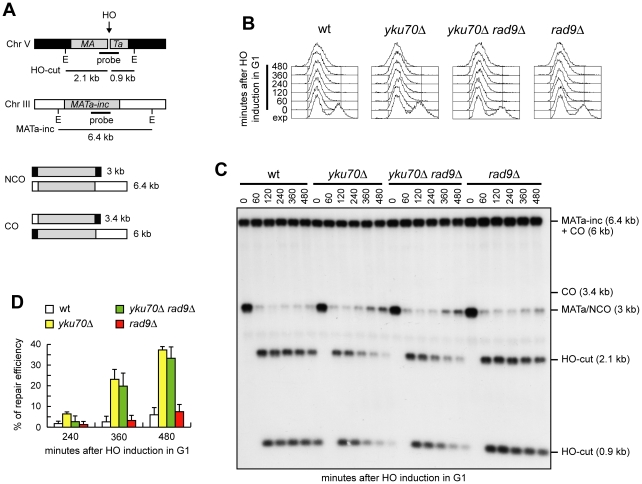
Generation of ssDNA bypasses Cdk1 requirement for noncrossover recombination. (A) In all the strains with the indicated genotypes, galactose-induced HO generates a DSB at a *MATa* DNA sequence inserted on chromosome V, while the homologous *MATa-inc* region on chromosome III cannot be cut by HO and is used as a donor for HR-mediated repair, which can generate both noncrossover (NCO) and crossover (CO) products. The sizes of EcoRI (E) fragments detected by the depicted probe are indicated. (B–D) Exponentially growing YEP+raf (exp) cell cultures were arrested in G1 with α-factor (time zero) and transferred to YEP+raf+gal in the presence of α-factor. (B) FACS analysis of DNA content. (C) Southern blot analysis of EcoRI-digested genomic DNA with the *MATa* probe depicted in A. (D) Densitometric analysis of the repair signals. Plotted values are the mean value ±SD from three independent experiments as in (C). See Materials and Methods for details.

When both ends of a DSB share homology with an intact DNA sequence, repair by Rad51-dependent recombination pathways leads to the formation of noncrossover or crossover products. We investigated whether generation of 3′-ended ssDNA can bypass Cdk1 requirement also in this process. To detect crossovers and noncrossovers at the molecular level, we used a haploid strain that bears two copies of the *MATa* sequence (Figure 6A) [28], [36]. One copy is located ectopically on chromosome V and carries the recognition site for the HO endonuclease, while the endogenous copy on chromosome III carries a single base pair mutation that prevents HO recognition (*MATa-inc*). Upon galactose addition, the HO-induced DSB can be repaired by Rad51-dependent HR using the uncleavable *MATa-inc* sequence as a donor. This repair event can occur either with or without an accompanying crossover (Figure 6A) with the proportion of crossovers being 5–6% among the overall repair events [28], [36]. We induced HO expression in α-factor-arrested cells that were kept arrested in G1 in the presence of galactose (Figure 6B). Galactose was maintained in the medium to cleave the HO sites that were eventually reconstituted by NHEJ-mediated DSB repair. The 3 kb *MATa* band resulting from recombination events that are not associated to crossovers re-accumulated in both *yku70Δ* and *yku70Δ rad9Δ* G1 cells, but not in wild type and *rad9Δ* G1 cells (Figure 6C and 6D). The repair efficiency in both *yku70Δ* and *yku70Δ rad9Δ* G1 cells was around 40% after 8 hours (Figure 6C and 6D), reaching 80–90% after 24 hours (data not shown). This finding indicates that the absence of Yku is sufficient for noncrossover HR events to take place despite of the low Cdk1 activity.

### Cdk1 requirement for crossover recombination

**Figure 7:**
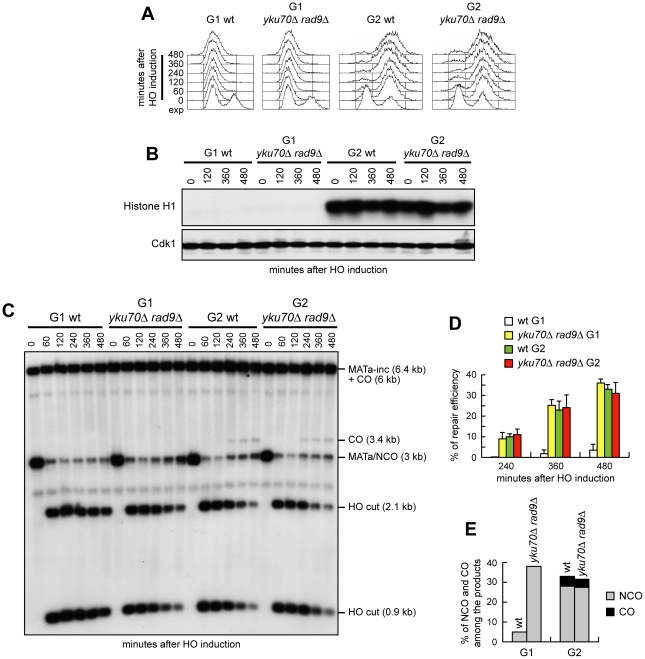
Generation of ssDNA does not bypass Cdk1 requirement for crossover recombination. Exponentially growing YEP+raf (exp) wild type and *yku70Δ rad9Δ* cells carrying the system described in Figure 6A were arrested at time zero in G1 with α-factor or in G2 with nocodazole, and transferred to YEP+raf+gal in the presence of α-factor or nocodazole, respectively. (A) FACS analysis of DNA content. (B) Cell samples of strains expressing fully functional Cdc28-HA were taken at the indicated times to assay Cdk1 kinase activity (top row) and to determine Cdk1 levels (bottom row) as in Figure 1F. (C) Southern blot analysis of EcoRI-digested genomic DNA as described in Figure 6. (D) Densitometric analysis of repair band signals (CO+NCO). Plotted values are the mean value ±SD from four independent experiments as in (C), enclosing that described in (B). (E) Densitometric analysis of CO versus NCO repair bands at 480 minutes from break induction. See Materials and Methods for details.

Interestingly, the 3.4 kb chromosomal band expected in the experiment above in case of crossover products was not detectable in any G1 cell culture (Figure 6C), suggesting a role for Cdk1 in promoting crossover outcomes that is different from its function in DSB resection. We then compared the products of interchromosomal recombination in G1- and G2-arrested wild type and *yku70Δ rad9Δ* cells (Figure 7A). As expected, Cdk1 kinase activity remained undetectable in all α-factor arrested cell cultures, whereas it was high in G2-arrested cells (Figure 7B). The overall DSB repair efficiency of G1-arrested *yku70Δ rad9Δ* cells was similar to that of G2-arrested wild type and *yku70Δ rad9Δ* cells (Figure 7C and 7D). However, while no crossover events were detectable in *yku70Δ rad9Δ* G1 cells, ∼4–5% of repair events were associated to crossovers in both wild type and *yku70Δ rad9Δ* G2 cells, as indicated by the appearance of the 3.4 kb crossover band (Figure 7C and 7E). Thus, *yku70Δ rad9Δ* G1 cells appear to be specifically defective in generating crossover products. This inability was not due to the absence of Yku and/or Rad9, because similar amounts of crossover products were detectable in wild type and *yku70Δ rad9Δ* G2-arrested cells (high Cdk1 activity) (Figure 7C and 7E). These results suggest that Cdk1 has a function in promoting crossover recombination that is independent of its role in DSB resection.

**Figure 8:**
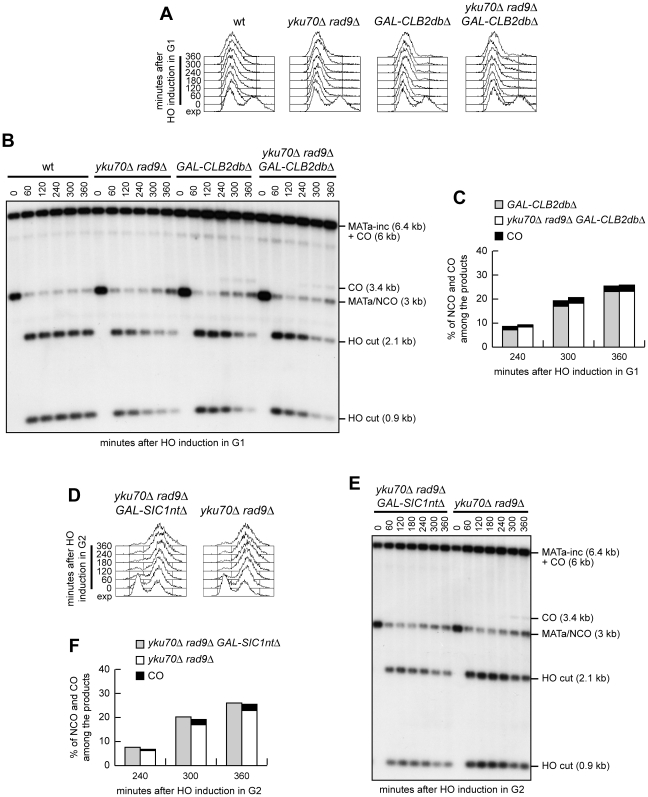
Ectopic Cdk1 activation allows crossovers in G1, whereas Cdk1 inhibition prevents crossover in G2. (A–C) Exponentially growing YEP+raf (exp) cultures of cells with the indicated genotypes and carrying the system described in Figure 6A were arrested at time zero in G1 with α-factor and transferred to YEP+raf+gal in the presence of α-factor. (A) FACS analysis of DNA content. (B) Southern blot analysis of EcoRI-digested genomic DNA as described in Figure 6. (C) Densitometric analysis. Plotted values are the mean value from two independent experiments as in (B). (D–F) Exponentially growing YEP+raf cells with the indicated genotypes and carrying the system described in Figure 6A were arrested at time zero in G2 with nocodazole and transferred to YEP+raf+gal in the presence of nocodazole. (D) FACS analysis of DNA content. (E) Southern blot analysis of EcoRI-digested genomic DNA as described in Figure 6. (F) Densitometric analysis. Plotted values are the mean value from three independent experiments as in (E).

If the inability to perform crossover recombination in G1 were due to the lack of Cdk1 activation, then ectopic expression of active Cdk1 should allow crossover recombination in G1, whereas Cdk1 inhibition should prevent crossover formation in G2. We then constructed wild type and *yku70Δ rad9Δ* strains carrying the system in Figure 6A and expressing a stable version of the mitotic cyclin *CLB2* under the control of the *GAL* promoter (*GAL-CLB2dbΔ*). This Clb2 variant forms active Clb2-Cdk1 complexes also during G1, because it lacks the destruction box, and therefore it is not subjected to B-type cyclin-specific proteolysis [37]. Strikingly, when both DSB formation and Clb2db*Δ* overproduction were induced in G1-arrested cell cultures by galactose addition (Figure 8A), crossover products became detectable in both *GAL-CLB2dbΔ* and *yku70Δ rad9Δ GAL-CLB2dbΔ* cells, whereas they were not present in wild type and *yku70Δ rad9Δ* cells under the same conditions (Figure 8B and 8C).

To assess whether Cdk1 inhibition prevented crossover formation in G2, we compared the products of interchromosomal recombination in G2-arrested *yku70Δ rad9Δ* and *yku70Δ rad9Δ GAL-SIC1ntΔ* cells (Figure 8D), the latter expressing high levels of a stable version of the Cdk1 inhibitor Sic1 (Sic1nt*Δ*) [35]. When both DSB formation and Sic1nt*Δ* overproduction were induced in G2-arrested cell cultures by galactose addition, crossover products accumulated, as expected, in *yku70Δ rad9Δ* cells, but they were undetectable in *yku70Δ rad9Δ GAL-SIC1ntΔ* cells (Figure 8E and 8F). Thus, Sic1-mediated Cdk1 inhibition prevents generation of crossover products in G2, whereas ectopic Cdk1 activation leads to crossover recombination in G1, supporting the hypothesis that Cdk1 activity is required to promote crossover HR events even when DSB resection is allowed by the absence of Yku and Rad9.

## Discussion

HR is highly coordinated with the cell cycle: it takes place predominantly during the S and G2 phases, when the presence of a sister chromatid provides a donor template and high Cdk1 activity promotes DSB end resection to expose ssDNA that is necessary to initiate HR [17], [18], [21], [30]. To study whether Cdk1 plays additional role(s) in HR, we asked whether generation of ssDNA at the DSB ends is sufficient to bypass Cdk1 requirement for HR. Because the lack of either Yku or Rad9 allows Cdk1-independent generation of 3′-ended ssDNA at DSB ends [24], [26], we investigated whether cells lacking Yku and/or Rad9 could repair a DSB by HR when Cdk1 activity is low. We found that DSB repair by SSA can take place in G1-arrested *yku70Δ* cells. The ability of these cells to carry out SSA in G1 depends on Cdk1-mediated generation of 3′-ended ssDNA at the DSB ends. In fact, the lack of Rad9 increases efficiency of both resection and SSA in *yku70Δ* G1 cells. Furthermore, Cdk1 inhibition prevents SSA in G2 wild type cells, but not in *yku70Δ rad9Δ* G2 cells, where DSB resection occurs independently of Cdk1. We also found that G1-arrested *yku70Δ* and *yku70Δ rad9Δ* cells can undergo interchromosomal recombination events that are not accompanied by crossovers. Thus, Cdk1 requirement for carrying out SSA and noncrossover recombination is bypassed by DSB resection, indicating that Cdk1 promotes these HR events essentially by regulating the resection step.

Rad52 is essential for both SSA and noncrossover recombination events, while only the latter require the assembly of Rad51 nucleoprotein filaments, which promote homologous pairing and strand exchange (reviewed in [1]–[3]). As the function of Cdk1 in DSB repair by SSA and noncrossover recombination is primarily the regulation of the resection step, neither Rad51 nor Rad52 appear to require Cdk1 activity to exert their biochemical activities.

Interestingly, although *RAD9* deletion was shown to allow MRX-dependent DSB resection in G2 cells that overproduced the Cdk1 inhibitor Sic1 [26], the lack of Rad9 did not increase DSB resection or HR-mediated DSB repair in G1 compared to wild type cells. Thus, although Rad9 provides a barrier to resection in *yku70Δ* G1 cells, its lack is not sufficient, by itself, to escape the inhibitory effect of Yku on DSB resection in G1. This finding is consistent with previous data showing that the resection block imposed by Yku is relieved in G2 [33], [34]. It also indicates that Rad9 prevents DSB resection in all cell cycle phases, but its inhibitory effect in G1 becomes apparent only in the absence of Yku.

Surprisingly, we found that G1-arrested *yku70Δ rad9Δ* cells are specifically impaired in the formation of crossovers by interchromosomal recombination. Expression of an activated form of Cdk1 allows crossover recombination in both wild type and *yku70Δ rad9Δ* G1 cells, whereas inhibition of Cdk1 activity in G2-arrested *yku70Δ rad9Δ* cells prevents crossover formation without affecting noncrossover outcomes. These findings are consistent with a role of Cdk1 in promoting crossover recombination that is independent of its function in DSB resection.

How does Cdk1 promote crossover outcomes? The choice between crossover and noncrossover is tightly regulated [38]. Meiotic recombination results frequently in crossovers [39], while DSB repair in mitotic cells is mostly not associated with crossovers [6]. An explanation of these differences could be that specific mechanisms limit crossovers during mitotic homologous recombination. Indeed, dissociation of the D-loop intermediates gives rise to noncrossover products, and this process is promoted by the helicases Srs2 and Mph1 [7], [28], [36], [40]. Furthermore, noncrossover outcomes can arise also from the dissolution of dHJ intermediates that requires the combined activity of the BLM/Sgs1 helicase, which drives migration of the constrained dHJs, and the Top3-Rmi1 complex, which decatenates the interlinked strands between the two HJs [7]–[9]. One possibility is that Cdk1 promotes crossover recombination by inhibiting proteins specifically involved in limiting crossover generation (i.e. Sgs1, Top3-Rmi1, Srs2 and Mph1). A similar mechanism seems to act during meiotic recombination, where proteins required for homologous chromosome synapsis have been proposed to antagonize the anti-crossover activity of Sgs1 [41]. However, none of the above anti-crossover proteins have been reported to undergo Cdk1-dependent inhibitory phosphorylation. On the other hand, crossovers arise from dHJ intermediate cleavage, which involves the resolvases Mus81-Mms4, Slx1-Slx4, Yen1 and Rad1-Rad10 (reviewed in [42]), suggesting that Cdk1 might promote crossover recombination by stimulating dHJ resolution. Consistent with this hypothesis, the Yen1 and Mms4 resolvases appear to be phosphorylated by Cdk1 [43], raising the possibility that they might represent Cdk1 targets in dHJ resolution. Further studies will be required to assess whether Cdk1-dependent phosphorylation of these proteins has a role in regulating crossover formation.

In conclusion, Cdk1 controls primarily DSB resection to allow SSA and noncrossover recombination, while crossover outcomes appear to require additional Cdk1-promoted events. As mitotic crossovers have the potential for deleterious genome rearrangements, their Cdk1-dependent regulation can provide an additional safety mechanism, ensuring that the rare mitotic recombination events accompanied by crossing over at least occur in S/G2, when a sister chromatid is available as appropriate donor.

## Materials and Methods

### Yeast strains

Strain genotypes are listed in Table S1. Strains JKM139, YMV86 and YMV45 were kindly provided by J. Haber (Brandeis University, Waltham, USA). Strains YMV86 and YMV45 are isogenic to YFP17 (*matΔ::hisG hmlΔ::ADE1 hmrΔ::ADE1 ade1 lys5 ura3-52 trp1 ho ade3::GAL-HO leu2::cs*) except for the presence of a *LEU2* fragment inserted, respectively, 0.7 kb or 4.6 kb centromere-distal to *leu2::cs* [32]. Strain tGI354 was kindly provided by G. Liberi (IFOM, Milano, Italy) and J. Haber [28]. To induce a persistent G1 arrest with α-factor, all strains used in this study carried the deletion of the *BAR1* gene, which encodes a protease that degrades the α-factor. Deletions of the *YKU70*, *RAD9*, *RAD51*, *RAD52* and *BAR1* genes were generated by one-step PCR disruption method. YMV86, YMV45 and tGI354 derivatives strains carrying a fully functional *CDC28-HA* allele at the *CDC28* chromosomal locus were generated by one-step PCR tagging method. A plasmid carrying the *GAL-CLB2dbΔ* allele was kindly provided by R. Visintin (IEO, Milan, Italy) and was used to integrate the *GAL-CLB2dbΔ* fusion at the *URA3* locus in the tGI354 derivative strains. Strain YLL3019, carrying the *GAL-SIC1ntΔ* allele integrated at the *URA3* locus, was obtained by transforming strain tGI354 *rad9Δ yku70Δ* with ApaI-digested plasmid pLD1, kindly provided by J. Diffley (Clare Hall Laboratories, South Mimms, United Kingdom). The *GAL-SIC1ntΔ* fusion was cloned into a *TRP1*-based integrative plasmid that was used to integrate the fusion at the *TRP1* locus in the YMV45 derivative strains. Integration accuracy was verified by Southern blot analysis. Cells were grown in YEP medium (1% yeast extract, 2% bactopeptone) supplemented with 2% raffinose (YEP+raf) or 2% raffinose and 3% galactose (YEP+raf+gal).

### Kinase assay

For Cdk1 kinase assays, protein extracts were prepared as described previously [44]. HA-tagged Cdk1 was immunoprecipitated with anti-HA antibody from 150 µg of protein extracts and the kinase activity in the immunoprecipitates was measured on histone H1 [45].

### DSB resection and repair

DSB formation and repair in YMV86 and YMV45 strains were detected by Southern blot analysis using an *Asp*718-*Sal*I fragment containing part of the *LEU2* gene as a probe. DSB end resection at the *MAT* locus in JKM139 derivative strains was analyzed on alkaline agarose gels as described in [24], by using a single-stranded probe complementary to the unresected DSB strand. This probe was obtained by in vitro transcription using Promega Riboprobe System-T7 and plasmid pML514 as a template. Plasmid pML514 was constructed by inserting in the pGEM7Zf EcoRI site a 900-bp fragment containing part of the *MATα* locus (coordinates 200870 to 201587 on chromosome III). Quantitative analysis of DSB resection was performed by calculating the ratio of band intensities for ssDNA and total amount of DSB products. DSB repair in tGI354 strain was detected as described in [28]. To determine the amount of noncrossover and crossover products, the normalized intensity of the corresponding bands at different time points after DSB formation was divided by the normalized intensity of the uncut *MATa* band at time zero before HO induction (100%). The repair efficiency (NCO+CO) was normalized with respect to the efficiency of DSB formation by subtracting the value calculated 2 hours after HO induction (maximum efficiency of DSB formation) from the values calculated at the subsequent time points after galactose addition.

## Supporting Information

Table S1 Yeast strains used in this study.
(DOC)
