# Supplementary material for: Distinct Cdk1 Requirements during Single-Strand Annealing, Noncrossover, and Crossover Recombination
Source: PLoS Genet. 2011 Aug 25;7(8):e1002263. doi: 10.1371/journal.pgen.1002263 (PMC3161966; doi:10.1371/journal.pgen.1002263)
Supplement: Table S1 — Yeast strains used in this study. (DOC) [file pgen.1002263.s001.doc]

# Supporting Information

**Table S1.** *Saccharomyces cerevisiae* strains used in this study.

| Strain | Relevant genotype | Source |
| --- | --- | --- |
| YMV86 | *ho hml****∆****::ADE1 mata****∆****::hisG hmr****∆****::ADE1 ade3::GAL-HO ade1 lys5 ura3-52 trp1 leu2::leu2-NATMX-HOcs* | 1 |
| YLL2756 | YMV86 *yku70****∆****::URA3* | This study |
| YLL3047 | YMV86 *CDC28-3HA::TRP1* | This study |
| YLL3048 | YMV86 *yku70****∆****::URA3 CDC28-3HA::TRP1* | This study |
| YMV45 *bar1****∆*** | *ho hml****∆****::ADE1 mata****∆****::hisG hmrΔ::ADE1 leu2::leu2(Asp718-SalI)-URA3-HOcs ade3::GAL-HO ade1 lys5 ura3-52 trp1 bar1****∆****::HPHMX* | 1 |
| YLL2912 | YMV45 *bar1****∆****::HPHMX yku70****∆****::NATMX* | This study |
| YLL2910 | YMV45 *bar1****∆****::HPHMX rad9****∆****::KANMX4* | This study |
| YLL2903 | YMV45 *bar1****∆****::HPHMX yku70****∆****::NATMX rad9****∆****::KANMX4* | This study |
| YLL3036 | YMV45 *bar1****∆****::HPHMX CDC28-3HA::TRP1* | This study |
| YLL3049 | YMV45 *bar1****∆****::HPHMX yku70****∆****::NATMX CDC28-3HA::TRP1* | This study |
| YLL3050 | YMV45 *bar1****∆****::HPHMX rad9****∆****::KANMX4 CDC28-3HA::TRP1* | This study |
| YLL3037 | YMV45 *bar1****∆****::HPHMX yku70****∆****::NATMX rad9****∆****::KANMX4 CDC28-3HA::TRP1* | This study |
| YLL2956 | YMV45 *bar1****∆****::HPHMX yku70****∆****::NATMX rad9****∆****::KANMX4 rad52****∆****::TRP1* | This study |
| YLL3043 | YMV45 *bar1****∆****::HPHMX yku70****∆****::NATMX rad9****∆****::KANMX4 rad51****∆****::TRP1* | This study |
| YLL3044 | YMV45 *bar1****∆****::HPHMX trp1::GAL-SIC1nt****∆****::TRP1* | This study |
| YLL3045 | YMV45 *bar1****∆****::HPHMX yku70****∆****::NATMX rad9****∆****::KANMX4 trp1::GAL-SIC1nt****∆****::TRP1* | This study |
| JKM139 *bar1Δ* | *MATa ho hml****∆****::ADE1 hmr****∆****::ADE1 ade1-100 leu2-3;112 lys5 trp1::hisG ura3-52 ade3::GAL-HO bar1****∆****::HPHMX* | 2 |
| YLL2892 | JKM139 *bar1****∆****::HPHMX yku70****∆****::URA3* | This study |
| YLL2962 | JKM139 *bar1****∆****::HPHMX rad9****∆****::KANMX4* | This study |
| YLL2978 | JKM139 *bar1****∆****::HPHMX yku70****∆****::URA3 rad9****∆****::KANMX4* | This study |
| tGI354 *bar1Δ* | *ho hml****∆****::ADE1 MATa-inc hmr****∆****::ADE1 ade1 leu2-3;112 lys5 trp1::hisG ura3-52 ade3::GAL::HO arg5,6::MATa::HPHMX bar1****∆****::TRP1* | 3 |
| YLL2954 | tGI354 *bar1****∆****::TRP1 yku70****∆****::URA3* | This study |
| YLL2980 | tGI354 *bar1****∆****::TRP1 rad9****∆****::KANMX4* | This study |
| YLL2970 | tGI354 *bar1****∆****::TRP1 yku70****∆****::URA3 rad9****∆****::KANMX4* | This study |
| YLL3019 | tGI354 *bar1****∆****::TRP1 yku70****∆****::NATMX rad9****∆****::KANMX4 ura3::GAL-SIC1nt****∆****-MYC-HIS::URA3* | This study |
| YLL3051 | tGI354 *bar1****∆****::TRP1 CDC28-3HA::URA3* | This study |
| YLL3052 | tGI354 *bar1****∆****::TRP1 yku70****∆****::NATMX rad9****∆****::KANMX4 CDC28-3HA::URA3* | This study |
| YLL3038 | tGI354 *bar1****∆****::TRP1 ura3::GAL-CLB2db****∆****::URA3* | This study |
| YLL3039 | tGI354 *bar1****∆****::TRP1 yku70****∆****::NATMX rad9****∆****::KANMX4 ura3::GAL-CLB2db****∆****::URA3* | This study |

**References**

1. Vaze MB, Pellicioli A, Lee SE, Ira G, Liberi G, Arbel-Eden A, Foiani M, Haber JE (2002) Recovery from checkpoint-mediated arrest after repair of a double-strand break requires Srs2 helicase. Mol Cell 10: 373-385.

2. Lee SE, Moore JK, Holmes A, Umezu K, Kolodner RD, Haber JE (1998) *Saccharomyces* Ku70, mre11/rad50 and RPA proteins regulate adaptation to G2/M arrest after DNA damage. Cell 94: 399-409.

3. Saponaro M, Callahan D, Zheng X, Krejci L, Haber JE, Klein HL, Liberi G (2010) Cdk1 targets Srs2 to complete synthesis-dependent strand annealing and to promote recombinational repair. PLoS Genet 6: e1000858.
